# Childhood Brain Development, the Educational Achievement Gap, and Cognitive Enhancement

**DOI:** 10.3389/fphar.2018.01142

**Published:** 2018-10-05

**Authors:** Fabrice Jotterand

**Affiliations:** ^1^Center for Bioethics and Medical Humanities, Medical College of Wisconsin, Milwaukee, WI, United States; ^2^Institute for Biomedical Ethics, University of Basel, Basel, Switzerland

**Keywords:** poverty, neuroscience, childhood poverty, neuroethics, cognitive enhancement, education, clinical ideal

## Abstract

Research on the effects of adversity on the brain of children initially encountered strong skepticism mainly due to the fear of stigmatization and the potential pathologizing of poverty as a disease. Despite initial resistance, an increasing body of work demonstrates a correlation between low socioeconomic status and brain development. This article will focus specifically on the impact of poverty (material, economic, and social) on childhood brain development and educational achievement. Some suggest that the use of cognitive enhancers in healthy students is perfectly acceptable and should be promoted to counterbalance the failure of traditional means to improve educational achievements. In this article, I critically assess the claim that a broad use of cognitive enhancers should be promoted and offer an alternative approach. The first section evaluates the neuroscientific facts and evidence of the impact of poverty on brain development and outlines some of the criticisms raised against the “neuroscience of poverty.” The second section focuses on the proposal made by Ray (2016) that promotes the use of cognitive enhancers as a means to address poor educational attainment. I criticize the basis of her argument and propose a different approach I call the clinical ideal. Subsequently, I provide some ethical pointers to allow an ethical and prudent use of cognitive enhancers in the educational setting. The main point of the article is not to reject prima facie the use of cognitive enhancers in socially disadvantaged students but rather provide a more nuanced approach.

## Introduction

One of the major factors contributing to disparities in early child development is poverty. In the United States, a country among the first world with the highest level of childhood poverty, poverty affects one in five children (Katsnelson, 2015) and studies have demonstrated that there is a connection between socioeconomic status and long term health outcomes, academic achievements and mental health. To understand the factors leading to these adverse outcomes, there has been an increased interest in understanding the effects of poverty on brain development in children (Hackman et al., 2010; Luby et al., 2013; Hair et al., 2015; Barch et al., 2016; Blair and Raver, 2016; Farah, 2017; Lipina and Evers, 2017). Childhood brain development is a complex and multilayered issue and poverty is only one factor among others. For instance, one study has focused on community violence exposure such as the witnessing of illegal drug use, street fights, or the hearing of gun shots. The findings revealed that exposure to neighborhood violence can affect brain regions associated with cognitive and emotional development in adolescents (Saxbe et al., 2018). It should be also noted that there is an increasing body of literature on neuroepigenetics that examines the multigenerational role of epigenetics in relation to poverty (Borghol et al., 2012; McGuinness et al., 2012). While certainly a domain to explore further with regard to childhood brain development, engaging this literature is beyond the scope of this article.

Research on the effects of adversity on the brains of children initially encountered strong skepticism mainly due to the fear of stigmatization and the potential pathologizing of poverty as a disease. Despite initial resistance, an increasing body of work demonstrates a correlation between low socioeconomic status and brain development. This article will focus specifically on the impact of poverty (material, economic, and social) on childhood brain development and educational achievement. Some suggest that the use of cognitive enhancers in healthy students is perfectly acceptable and should be promoted to counterbalance the failure of traditional means to improve educational achievements (Schwarz, 2012; Ray, 2016). In what follows, I critically assess the claim that a broad use of cognitive enhancers should be promoted and offer an alternative approach. The first section evaluates the neuroscientific facts and evidence of impact of poverty on brain development and outlines some of the criticisms raised against the “neuroscience of poverty.” The second section focuses on the proposal made by Ray (2016) that promotes the use of cognitive enhancers as a means to address poor educational attainment. I criticize the basis of her argument and propose a different approach I call the *clinical ideal*. Subsequently, I provide some ethical pointers to allow an ethical and prudent use of cognitive enhancers in the educational setting. It should be noted that the main point of the article is not to reject prima facie the use of cognitive enhancers in socially disadvantaged students but rather provide a more nuanced approach.

## The Neuroscience of Poverty

### Neuroscientific Facts and Evidence of Impact

The intention of this section is not to provide a comprehensive analysis of the neuroscientific evidence of the impact of poverty on neural development in children. However, a few definitions and neuroscientific facts are necessary to the subsequent analysis.

For the sake of clarity, and to capture the multidimensional nature of the phenomenon we commonly call poverty, I will use the terminology of socioeconomic status instead (SES) in the rest of the paper. Poverty denotes a state of affairs that is often interpreted as related to material considerations whereas SES indicates a multilayered concept that includes material (access to resources), economic (standards of living) and social (security, inclusion, social class) circumstances (Lipina and Evers, 2017). In addition, it is important to stress that generalizations about “poor vs. rich environments” should be avoided in discussions about the impact of poverty on brain development in children (Lipina and Evers, 2017). There are various influences that determine neural development and it is essential to contextualize the nature of the factors at play within particular social settings. To this end, Lipina and Evers (2017) provide a comprehensive and helpful list of protective and risk factors based on current literature on developmental psychology and cognitive neuroscience. They are: (1) prenatal maternal health; (2) perinatal health; (3) quality of early attachment; (4) level of stress at home and in school; (5) quality of parenting; (6) early cognitive and learning stimulation at home and educational contexts; (7) mental health of parents and teachers; (8) developmental disorders; (9) financial stressors on the family; (10) access to health and social services; (11) lack of social mobility; (12) social, political, and financial stress; (13) family, social, and cultural expectations about child development; and (14) natural disasters (Lipina and Evers, 2017). The successful implementation of strategic interventions to address the effect of low SES on children, especially brain development, will depend on the ability to develop a wide-ranging approach that takes into account the aforementioned factors which cannot be discussed comprehensively in this paper.

The examination of the effect of poverty on early brain development is a relatively new research focus in neuroscience. In early 2000, a research team led by cognitive neuroscientist Martha Farah began investigating the connection between SES, health outcomes, academic achievement, and mental health (Katsnelson, 2015). The “neuroscience of poverty” or the “neuroscience of socioeconomic status” is still not well understood and has encountered some resistance in its early stages due to the potential pathologizing of poverty as a “brain disease” or stigmatizing of poor children as “sick” (Katsnelson, 2015). Despite the early skepticism, Farah’s team published in 2005 an article in which they argued that there is an association between SES, cognitive ability and academic achievement in childhood and beyond (Noble et al., 2005). Since then, many other studies have been published that confirm the effects of poverty on childhood development such as its effects on mental, emotional, and behavioral health (van Goozen et al., 2008; Hackman et al., 2010); on structural brain development (Lawson et al., 2013; Luby et al., 2013; Brito and Noble, 2014; Barch et al., 2016; Saxbe et al., 2018); and on academic achievement (Hair et al., 2015). Some studies have demonstrated that children living in a disadvantaged socioeconomic environment “have poorer cognitive outcomes and school performances as well as higher risk of antisocial behavior and mental disorders” (Luby et al., 2013; see also Yoshikawa et al., 2012; Lipina and Evers, 2017). Other studies have confirmed neuroanatomical differences such as smaller hippocampal gray matter volumes and smaller amygdala (involved in emotions, memory, motivation, decision making, speech, and attention) among children from lower income backgrounds (Hanson et al., 2011; Jednoróg et al., 2012; Noble et al., 2015).

### Critical Perspectives (Yoshikawa et al., 2012)

While there is evidence that SES will impact brain development in children, some people have strong reservations about the validity of these findings. Yoshikawa et al. (2012) for instance, question whether poverty “causes negative outcomes.” In order to substantiate their claim, they raise two objections. First, they emphasize that “family poverty is complexly intertwined with a large number of what some researchers refer to as a poverty co-factors” (Yoshikawa et al., 2012, 273). In other words, there are determinants in prior generations that impact how poverty affects children. For instance, teen parenting and low academic attainment increase the chance of adolescent parents to raise children in poverty. Yoshikawa et al. (2012) also mention genetic factors and exposure to circumstances or contexts such as bad schools, depressed neighborhoods and difficult access to healthy nutrition (food desert). Second, they mention the challenge, in studies, to “employ research designs and statistical analyses that permit rigorous causal inference” (Yoshikawa et al., 2012, 274). The authors stress the importance of “the need of and logic of accounting” for the selection of factors in determining the effects of poverty (i.e., income) on brain development in children.

Another set of criticisms has been raised by Wax (2017) in a recent article. Her main criticism is that current neuroscientific research does not provide any useful information to develop policies and implement interventions that will address the effects of poverty on child brain development. As she points out, “neuroscientific research currently yields no information for shaping policy and designing effective interventions to address poverty and inequality and its associated consequences. Nor will it likely alleviate those problems in the foreseeable future” (Wax, 2017, 3). Wax offers two main reasons to support her assertion. First, there is a lack of evidence that neurological abnormalities and behavioral deficits can be determined by “innate [or] environmental causes of brain characteristics” (Wax, 2017, 3). Second, in her view, neuroscience does not offer any better insights than knowledge produced by other fields such as cognitive and behavioral psychology with regard to how society should address poverty and its adverse effects on child brain development (Wax, 2017).

The validity of these criticisms cannot be evaluated in this article due to its limited scope. As stated in the introduction, the focus here is how to tackle disparities in cognitive development among healthy children from socially disadvantaged contexts and whether the use of cognitive enhancers is an acceptable, morally and practically, means. To this end, I will now turn to a recent proposal made by Ray (2016) that stipulates the necessity, as an issue of social justice, of using stimulants in socially disadvantaged students.

## Cognitive Enhancers and the Educational Gap

### The Call for the Use of Cognitive Enhancers

In the last few years, there has been an increased focus in the neuroethics literature concerning the ethical implications of the use of cognitive enhancers in children or young adults without any behavior or cognitive disorders. Drugs such as Provigil have been used among executives and military pilots to increase alertness (Arrington, 2008; Talbot, 2009) whereas Adderall has been used to increase capabilities in academic performances in healthy individuals (Schwarz, 2012). Following this train of thought, cognitive enhancers are now considered as a potential means to compensate for disparities in cognitive development due to low SES. The thrust of the argument goes as follows: disadvantaged students from lower socioeconomic background did not have the same opportunities compared to students from wealthier contexts. Hence, we have an obligation, as a matter of social justice, to help those worse off using social and medical means such as cognitive enhancers.

Ray (2016) is a proponent of the use of stimulants to address the disadvantages some children are facing. She argues, in very pragmatic terms, that “we have to be willing to consider stimulants as an option because we are not correcting students’ disadvantages in other, more traditional ways” (Ray, 2016). Due to the failure to allocate more resources in less favored school districts and the apathy in promoting traditional means to address disparities, she points out that “[a]ddressing the problem of poor schools with increased funding, better training and pay for teachers, and updated teaching resources are all ways that could improve poor, underperforming schools, and make stimulants not needed (or less needed), but are all avenues that we as a society have decided that we are unwilling to pursue” (Ray, 2016, 32). Whether “we as a society” have decided not to pursue traditional means is debatable, at least a more nuanced critique should be offered as the issue is very complex. But this is a debate that is not the focus of my analysis.

That said, while she adopts a pragmatic approach, her argument relies strongly on questions pertaining to social justice, a highly debated philosophical concept. She argues that when factors that, to some extent, determine opportunities are unjustly distributed and affect children, other ways to help the worst off should be considered, stimulants included. In order to support her position, Ray has to demonstrate the validity of two major claims: (1) the use of cognitive enhancers to address disparities in cognitive development is not within the scope of therapy (children affected by poverty do not have a diseased brain), and (2) access to cognitive enhancers is an issue of social justice, or what she calls “opportunity maintenance,” and therefore students socially disadvantaged are entitled to their access and use. In the rest of this section, I assess these claims by outlining Ray’s conceptualization of the therapy-enhancement distinction in term of a “theory of just health care” and her concept of opportunity maintenance. I conclude the section by offering a critique and argue that Ray’s analysis is based on a logical and categorical mistake.

### The Therapy – Enhancement Distinction and Just Health Care

Rays starts her analysis of the therapy-enhancement distinction by a surprising claim. She argues that “the treatment/enhancement distinction is a *theory of just health care*…[t]reatement is characterized as medical interventions necessary to fend off or cure disease…enhancement is characterized as medically unnecessary interventions meant to improve upon normal functioning” (Ray, 2016, 32, italics mine). However, I will argue that the claim that the therapy-enhancement distinction is an issue of justice misconceptualizes the nature of the therapy-enhancement distinction. To help understand how it is the case, let’s turn to the work of Chadwick (2008) and Agar (2014). Both provide descriptions of enhancement beyond the mere limitation of the use of medical resources (aka “medically unnecessary interventions”) as characterized by Ray (see Jotterand, 2017 for a comparative table).

To evaluate whether the use of stimulants in socially disadvantaged students is morally acceptable it is imperative not to limit discussions to “just health care.” The danger is to politicize the debate without understanding conceptually what enhancement entails. According to Chadwick (2008) enhancement can be understood qualitatively (improvement view – to enhance is to make better), or quantitatively (additionality view – to enhance A is to add to, or exaggerate, or increase A in some respect) which focuses on either analyzing the criteria necessary to determine what is considered an improvement or on specific capacities for moral evaluation. Agar, on the other hand, offers two ways to conceptualize enhancement under the terminology of objective ideal and anthropocentric ideal. In the former case, the greater enhancement a technology produces the more valuable it is, whereas in the latter, enhancement is evaluated based on a balance between the intrinsic and instrumental value of an enhanced capacity. Other definitions have been advanced such as the clinical ideal (Jotterand, 2017). In this case, enhancement is assessed according to whether it improves the quality of life of an individual with mental impairment – more on the clinical ideal later in the paper. The main point in listing these definitions is to underscore that the issue of justice might be a question to consider carefully but to bypass key points such as the prudential value of enhancement, how certain values can be derived relative to human standards, or how enhancement should be evaluated with regard to quality of life is conceptually problematic and pragmatically ill-advised.

Ray’s analysis focuses almost exclusively on the question of the allocation of medical resources because in her view “the treatment/enhancement distinction does not account for the idea that individuals can be functioning properly but still be entitled to medical resources for well-being purposes” (Ray, 2016, 33; as I will outline in a subsequent section the concept of the clinical ideal addresses Ray’s concern without politicizing the issue). Central to her exploration of the issue is whether socially disadvantaged students are entitled to the use of cognitive enhancers as a matter of social justice. While I am sympathetic to the issue of social justice with regard to education, and health care, to name a few, and to making sure that enhancement technologies are not restricted to the best well off, it seems ethically problematic to ignore the more fundamental question of whether the use of stimulants is indeed a prudent way to tackle the issue of disparities in cognitive development and academic achievement.

First, many arguments advanced against the use of cognitive enhancers are not issues of just access to specific interventions that could enhance physical, mental or behavioral capacities. Even the ability to pay for such interventions would not make them, in principle, ethically acceptable. It is rather the nature of specific interventions and the means to achieve particular goals in the context of medical practice and the social milieu that may make enhancement ethically problematic. In addition, some enhancement interventions could be used for therapeutic purposes (Jotterand, 2017) and therefore do not raise issues of justice but rather become a matter of what benefits the patient – of course the issue of access will always be a concern but this is true of any intervention. It follows that the issue of the use of stimulants in the therapy/enhancement distinction is not a question of access and affordability since these drugs are relatively inexpensive.

Second, evidence demonstrates that poverty may affect structural brain development in socially disadvantaged students which can result in lower academic achievements (Lawson et al., 2013; Luby et al., 2013; Brito and Noble, 2014; Barch et al., 2016). I recognize the danger of using neuroscientific knowledge that could potentially stigmatize these children as having a “diseased brain.” However, in order to justify the use of stimulants and stir away from the therapy-enhancement debate, Ray contends that there is an obligation “to assist people with non-disease conditions.” She adamantly stresses that the use of stimulants aims at addressing *social deficits not biological deficits* because of the “abnormal social health” of some children who should have equal opportunities to good education. It is certainly the case that we have, as a society, an obligation to provide better educational opportunities to underprivileged children but intervention at the biological level to manage a social deficit undermines the need to reflect why we have come to the point of using “whatever” means. Technological expediency is never a solution to address inherently human and social problems and undermines the responsibility of citizens to engage in public debates about the issue. In addition, the argument that these children are not “sick,” that is, do not manifest structural brain development abnormalities, does not reflect neuroscientific data acquired through many studies as pointed out earlier. It does not mean that we can deduce a direct causation between neural abnormalities and lower academic achievements as more research on the topic is necessary before any conclusions can be drawn between the relation of SES and brain structure and function. However, it would be misguided to simply ignore the body of research on the topic. Farah rightly points out that

“a substantial body of research has revealed association between SES and brain structure and function. However, it would be premature to make any grand generalizations about a brain signature and SES or poverty…The specific ways in which neuroscience will contribute to understanding SES in the future are difficult to anticipate…Neuroscience can be expected to illuminate the processes by which SES becomes associated with a wide range of important life outcomes and to suggest ways of improving outcomes for people of low SES” (Farah, 2017, 62).

The important point here is that neuroscientific knowledge could in the future assist design interventions to aid people with low SES and help them achieve better academically. However, if atypical brain structure and function does not fall into the category of “abnormal” in the light of evidence of low academic achievement or behavioral problems, the translation of the neuroscientific data into interventions will face steep challenges. In short, social deficits can have biological and social causes. Both set of considerations must be included in potential interventional strategies.

Third, Ray does not provide a clear analysis as to why the language of treatment is inappropriate in her proposal – other than say that the treatment-enhancement distinction does not account for well-being. I will not elaborate further in this section as the next one will address this point more fully in relation to the concept of “opportunity maintenance.”

### Opportunity Maintenance

The inappropriateness of treatment-therapy language regarding the promotion of stimulants as a potential means to deal with social disadvantages is, according to Ray, that the target of interventions is social deficits and not biological deficits. She argues that well-being is not part of the therapy-enhancement debate and therefore, since stimulants would enhance the quality of life of socially disadvantaged students, these interventions lie outside biological considerations. However, embedded in her definition are medical assumptions about the student population under consideration. For instance, she claims that “the goal of opportunity maintenance is to make undesirable environments have less control over the futures open to disadvantaged children and to explore ways – medical and/or social – to create new opportunities for healthy lives” (Ray, 2016). Implicit to her definition of opportunity maintenance is the idea that medical means will be able to improve the quality of life of some individuals on the assumption that boosting cognitive capacities (due to diminished cognitive capacities) will lead to better academic achievement. In addition, she refers to “new opportunities for healthy lives,” which seems to imply achieving a healthy state based on the recognition of a suboptimal state of health. Ultimately, she contends, the goal is to minimize the effects of social deficits “without assigning pathologies” (Ray, 2016). But Ray is making a categorical and logical mistake. The logical mistake is the assumption that a social deficit can necessarily be addressed through biological interventions. In no way will a stimulant create new opportunities if the social environment is not conducive to better educational outcomes. Access to good education, i.e., the content of educational material, competent teachers, and adequate resources, is not a question of cognitive capacities but access. Of course, if there are deficiencies in cognitive abilities some drugs might help but it would means diagnosing a condition, which is precisely what Ray wants to avoid. This train of thought leads to a categorical mistake. Cognitive enhancers will only affect biological dimensions of human experience. How social deficits could be resolved as the result of such interventions will mostly depend on individuals and the type of support and resources available to them. Conversely, changing the social context of individuals might positively affect their health but if an underlying condition is present, changes of the social milieu will have limited, if any, effect. The categories of “social” and “biological” ought to be clearly defined and distinguished, and the potential intersection of how particular interventions might influence each other conceptually explained.

Overall, Ray’s proposal to promote the use of stimulants to alleviate the effects of social determinants on academic achievement and to create opportunities is conceptually problematic and empirically confused. There is enough evidence that low SES affects brain development (correlation not causation) and consequently could affect academic achievement. Unless we recognize the relevance of neuroscientific information, the issue will be limited to resource allocation without the acknowledgment that poor environment impacts brain development. Ultimately such a strategy will negatively impact those who have been affected. If individuals suffer from an underlying biological condition (biological deficit) that can be addressed, restricting the debate to social deficits is detrimental to these individuals. It might be a risky road to take due to the potential of stigmatization, but researchers have established the association between SES and brain structure and function.

## The Clinical Ideal and Cognitive Enhancers

In the light of these considerations, I would like to suggest an approach that avoids the pitfalls outlined in the previous section. What I am proposing is a slight alteration of the concept of the *clinical ideal* I developed elsewhere (Jotterand, 2017) in relation to mental impairment and cognitive enhancers. It is recognition that a neural explanation of cognitive deficits that have social implications ought not to be excluded outright. For instance, Farah has suggested that “neural explanations should be considered alongside structural societal explanations and that, in some cases, neural explanations may be uniquely informative” (Farah, 2017, p. 57). It is this dual dimension that needs to be exploited without falling into an instrumentalization of neuroscientific knowledge for social engineering. Human brain development is a complex process at the intersection of biological development and environment factors interacting with each other – what is commonly called brain plasticity. Brain plasticity is the phenomenon that explains how environmental factors constantly shape neural pathways and therefore the exposure to violence, stress, malnutrition, and poor health care, to name a few, should be recognized as potential damaging contributors to brain development in children, which can lead to poor academic performance. It is important to stress that individuals exposed to these challenges do not have “an immutable and irreversible deficit condition” (Lipina and Evers, 2017, 7). We should adamantly reject the view that growing up in poverty or being exposed to malnutrition have irreversible consequences as sometimes reported by some media (Stromberg, 2013; Graham-Harrison, 2014).

### The Clinical Ideal

Before we can focus on how the concept of the clinical ideal can help frame the issue of the use of cognitive enhancers in the education setting, I will provide the following working definition:

“[The clinical ideal] assigns value to enhancement if it enhances the physical, mental, and social capacities and the overall quality of life of an individual with mental impairment (baseline disabled) in the context of life (contextual standards). The clinical ideal facilitates the introduction of the notion of enhancement in the clinical language without the need to contrast it or oppose it to the notion of therapy. It limits its scope of considerations to the life of a particular individual whose quality of life and well-being could be improved by cognitive enhancers” (Jotterand, 2017, 418–19).

Usually therapy implies the restoration, in various degrees, of the functions of a biological entity impacted by the deleterious effects of disease. In other words, there are criteria that define the scope of medical practice and establish baseline standards to determine whether a clinical intervention is justified. The clinical ideal does not require a clear demarcation of the scope of therapy vs. the scope of enhancement. It recognizes the dual effects of some drugs and devices whereas some therapeutic interventions might have enhancing side effects. Hence, whether or not an intervention falls into the category of therapy or enhancement is irrelevant. The clinical ideal merges the two concepts (therapy and enhancement) and points to the well-being and the improvement of the quality of life of the individual in the context of his or her *current* life. However, the notions of quality of life and well-being in the health care setting are not established by threshold criteria. They depend on various factors such as the nature of the disease and the side effects of treatment; the ability of the patient to perform basic everyday activities; how a patient experiences happiness, suffering, and pleasure; and the patient’s level of independence and privacy as well sense of dignity (Lo, 2000, 30) The above framework avoids a false dichotomy between enhancement and therapy that occurs in some conceptualizations of enhancement as reflected by the work of Ray.

In addition, another clarification is warranted. The clinical ideal presupposes a distinction between enhancement #1 and enhancement #2 (Jotterand et al., 2015; Jotterand, 2017). In enhancement #1, individuals are healthy and are considered non-disabled at baseline. They display a normal range of human capacities and the bodies of these individuals do not require any medical attention to achieve a state of homeostasis. Interventions in unhealthy individuals within this group means therapy aimed at restoring baseline. Conversely, in enhancement #2 individuals at baseline have a cognitive deficit which limits their ability to function normally (i.e., adequate educational achievement in the context of this article) but otherwise they are healthy. This signifies that the target of the clinical ideal is healthy individuals who function below optimal capabilities based on neurological criteria that are manifested in differences in cognitive development. It is important to note that notions of normality, health, or disability are evolving concepts based on a complex web of biological, environmental, social and personal factors that shape how one understands health and the expectations about how to achieve a state of health. Therefore, while there is a subjective dimension in establishing standards, the practice of medicine requires the delineation of standards (i.e., normal range of human capacities) within the confines of human biology.

The improvement of quality of life and well-being of individuals within the context of their *particular* life is the third key element. To avoid the promotion of cognitive enhancers regardless of the careful attention to one’s circumstances, their use ought to be considered only if they increase quality of life and well-being. Needless to point out that these two notions are subjective and prone to variable interpretations (see Jotterand, 2017 for a more in-depth analysis). Hence, the model proposed is highly individualized which allows a fine-grained evaluation of the potential justification of the use of stimulants in the educational context. For instance, a teenager might be willing to take cognitive enhancers but before allowing such a step, parents and prescribers should determine whether the lack of educational achievement is due to environmental factors, neurological deficits, the lack of engagement on the part of the student or a mix of all these factors. In other words, the clinical ideal does not outright condone or support the use of cognitive enhancers in the educational and social milieus. Rather, it requires an evaluation of the level of enhancement in relation to the degree of quality of life improvement within the context of the individual requesting the intervention.

### Ethical Considerations

In principle, then, the use of cognitive enhancers could be justified if the above caveats are carefully considered. However, there are ethical considerations related to the use of neuroenhancers that should not be overlooked. Ilina Singh and Kelly J. Kelleher have provided an excellent overview of key areas of ethical concerns with regard to the use of neuroenhancers in young people (Singh and Kelleher, 2010). First, there is the issue of safety and side effects. Currently, there are no studies examining the long terms effects of stimulants on healthy subjects and therefore only drugs with low abuse potential should be prescribed and only short-term use should be warranted until more data is collected about the safety and effects of cognitive stimulants, their potential addictive power, and the length individuals should use cognitive enhancers in the educational setting. Furthermore, particular attention should be on how stimulants could affect long term the developing brain of children and adolescents. Second, the process of consenting young people needs to be clearly delineated as they are a vulnerable population subject to manipulation and abuse. Parents should be well informed and consented, and the assent/consent, depending on age and maturity of adolescents under 18, should be sought. Individuals above 18 should be consented without coercion but extra precaution should take place as the brains of young adults are still developing. Consent does not mean that these individuals fully understand the implications of the intake of stimulants. There might be a pressure to succeed in school and therefore a willingness to take risks may be higher than usual. For this reason, educational materials that meticulously outline the potential risks and benefits of neuroenhancers should be available and age-appropriate. Third, considering how certain drugs can impact personal identity, the prescription of neuroenhancers should be preceded by an evaluation of the recipient’s self-perception and moral understanding. Moreover, following the intake of the drug a tracking of the potential changes should be documented. Finally, Singh and Kelleher point out that criteria for equal access (availability and cost) should be established, as well as the determination of clear boundaries to avoid social coercion. Potentially, the availability of neuroenhancers could create an incentive for parents to push their children to excel in high school to get into the best colleges. Likewise, college students could be motivated to use stimulants to achieve higher grades but potentially be jeopardizing their health. In addition, although beyond the scope of this article, other alternatives to mitigate the impact of poverty on cognition and learning abilities should be carefully explored and considered under the right circumstances if such techniques are safe and efficacious (Hildebrandt et al., 2017; Zelazo et al., 2018).

## Concluding Remarks

As the use of cognitive enhancers is becoming increasingly widespread, it is imperative to proactively anticipate their potential misuses and abuses. As stated in the paper, prima facie prescription or the use of cognitive enhancers in educational settings is not inherently inappropriate. In some cases, it might be adequate, if not required, to use stimulants to help students perform well in school. Such moves, however, must be guided by strict boundaries and a clear explanation concerning the rationale justifying the enhancement of cognitive abilities in socially disadvantaged students. In addition, neuroscientific knowledge must provide the basis by which the use of stimulants is justified. Simply looking at the lack of academic achievements as a justification for the prescription of drugs such as Adderall or Modafinil is imprudent even though it might remove the potential of stigmatization or the pathologizing of poverty. Neuroscience might have unique insights that could benefit individuals from socially disadvantaged backgrounds.

Technology is a blessing and a curse. A blessing because it can assist in improving the human condition but also a curse when we, as a society, stop reflecting on how human agency can be part of the solution of social problems such as access to good education, and seek technological solutions instead. Discerning when technology is appropriate to use to address a social problem requires going beyond pragmatic considerations and including a careful reconsideration of what it means to live in communities and to flourish as human beings.

## Author Contributions

The author confirms being the sole contributor of this work and approved it for publication.

## Conflict of Interest Statement

The author declares that the research was conducted in the absence of any commercial or financial relationships that could be construed as a potential conflict of interest.
